# Enhanced glomerular thrombosis in pronated animals with ARDS

**DOI:** 10.1186/s40635-025-00747-7

**Published:** 2025-03-20

**Authors:** Silvia Marchesi, Elin Lundström, Elin Lindström, Jonas Ödmark, Mark Lubberink, Håkan Ahlström, Miklós Lipcsey

**Affiliations:** 1 Hedenstierna Laboratory, Department of Surgical Science, Uppsala University, Uppsala, Sweden; 2 Department of Intensive and Perioperative Care, Skåne University Hospital, Malmö, Sweden; 3 Department of Medical Science, Lund Universitet, Lund, Sweden; 4 Anesthesiology and Intensive Care, Department of Surgical Science, Uppsala University, Uppsala, Sweden; 5 Radiology, Department of Surgical Science, Uppsala University, Uppsala, Sweden; 6 Molecular Imaging and Medical Physics, Department of Surgical Sciences, Uppsala University, Uppsala, Sweden; 7 Medical Physics, Uppsala University Hospital, Uppsala, Sweden; 8 Antaros Medical, Mölndal, Sweden

**Keywords:** Prone position, Ventilation, Acute respiratory distress syndrome, Abdominal inflammation, Abdominal perfusion, Abdominal edema, Renal perfusion, Glomerular thrombosis

## Abstract

**Background:**

Prone positioning is part of the management of acute respiratory distress syndrome (ARDS) and has been demonstrated to successfully improve the ventilation–perfusion match and reduce mortality in patients with severe respiratory failure. However, the effect of pronation on other organs than the lungs has not been widely studied. This study aimed to compare abdominal edema, perfusion and inflammation in supine and prone positioning in a porcine ARDS model.

**Methods:**

Seventeen piglets were randomized into two groups: a supine group (*n* = 9) and a prone group (*n* = 8). Both groups received endotoxemic infusion and were observed for 6 h. Three animals per group underwent positron emission tomography–magnetic resonance imaging (PET–MRI) for imaging acquisition. Hemodynamic and respiratory parameters were recorded throughout the protocol. Inflammation was assessed by measuring cytokine concentrations in blood, ascites and the abdominal organs’ tissue. The edema in abdominal organs was assessed by wet–dry ratio and pathophysiological analysis of tissue samples and by MRI and PET measurements from volumes of interest (VOIs) delineated in abdominal organ in MRI and PET images. The abdominal organs’ perfusion was also assessed by MRI and PET measurements.

**Results:**

The prone group had a faster CO_2_ washout and needed a lower positive end-expiratory pressure to maintain the desired oxygenation. In the prone group duodenal edema was lower (measured with wet–dry ratio) and renal perfusion, by both MRI and PET measurements, was lower than half compared to the supine group (MRI, perfusion fraction, *f*: supine group 0.13; prone group 0.03; *p-value* 0.002. PET Flow: supine group 1.7; prone group 0.4 ml/cm^3^/min; *p-value* 0.002). In addition, the histopathological samples of the kidneys showed a higher incidence and extent of glomerular thrombosis in the prone group.

**Conclusions:**

In a porcine ARDS model, prone positioning was associated with enhanced glomerular thrombosis and low renal perfusion.

**Supplementary Information:**

The online version contains supplementary material available at 10.1186/s40635-025-00747-7.

## Introduction

For 30 years, prone positioning has been used to manage acute respiratory distress syndrome (ARDS) [1] and it has been linked to improved oxygenation, better gas distribution, and optimal ventilation–perfusion match [2]. Several trials have found a survival benefit deriving from pronation (reported to be from 10 to 17%) in patients with severe ARDS (PaO_2_/FiO_2_ < 100) [1, 3]. In this subpopulation, the timing of pronation is paramount to increase survival: early pronation results in a lower 28-day and 90-day mortality of 16 and 17%, respectively [4].

During the recent SARS-CoV-2 pandemic, the use of pronation has increased dramatically [5]and it was also applied to non-invasively ventilated patients, resulting in a lower intubation rate [6], but not affecting mortality [7, 8].

There are no absolute contraindications for pronation, except for unstable spinal fractures [9].

The most common prone position complications are pressure sores and facial edema [9], but severe desaturation, barotrauma, peripheral nerve injuries, and hypotension have also been reported [10]. Overall, very few studies have examined the effects of prone positioning on organs other than the lungs and most of the available data are incidental findings in lung-focused studies.

The effect of pronation on circulation was investigated, showing no adverse effects on hemodynamics when the maneuver was compared to the use of positive end-expiratory pressure (PEEP) [11] in supine patients. A slightly increased intra-abdominal pressure (IAP) has been described in some settings [12, 13], whereas other reports could not demonstrate that IAP was affected by the body position [14]. A reduction in the renal fraction of the cardiac output that did not affect the glomerular filtration rate (GFR) and renal function was reported in a small study [15]. During the pandemic, one study linked pronation to a higher incidence of deep vein thrombosis [16], but no other studies confirmed this association.

This study aims to investigate the impact of prone positioning on the perfusion, edema and inflammation of multiple abdominal organs in an ARDS animal model.

## Materials and methods

This study was approved by the Animal Research Ethical Committee of Uppsala University (Dnr 5.8.18-08592/2019).

Piglets from 3 to 4 months of age were used in the study. Experiments were performed during the daytime at the Hedenstierna Laboratory, Uppsala University Hospital. All piglets came from a certified farm, and they fasted, with free access to water, from the night before the experiment. They were randomized into a supine and a prone group (for the randomization method, see the Statistical analysis section).

In both groups, an endotoxin infusion was administered to induce mild to moderate ARDS.

An arterial line was placed in a neck artery to measure arterial pressure and collect blood samples; a central venous catheter placed in the jugular vein was used as an administration port for anesthetic drugs, endotoxin and fluids; and a Swan–Ganz catheter was placed in the pulmonary artery through the same jugular vein to measure wedge pressure. A urinary catheter was placed to collect urine and to measure intra-abdominal pressure.

For a detailed description of the preparation phase, see Appendix 1.

Hemodynamic parameters were continuously monitored, as well as respiratory parameters.

In the supine group, the animals were left in a supine position like that employed during the preparation phase; the animals in the prone group were positioned prone on the table from 30 min after the preparation and maintained in the same position till the end of the observation (6 h).

### Protocol

After preparation, a 30-min rest period was applied. Baseline measurements were then taken, and the animals were randomized to one of the two intervention groups (supine position group and prone position group, hereafter referred to as the Supine group and the Prone group, respectively).

Animals randomized to the Prone group were placed in the sphinx position using pillows to slightly elevate the head and the upper thorax. Animals randomized to the Supine group were maintained in the same position as during preparation. The observation time was started at time of the endotoxin infusion (Sigma E-coli O111:B4) at a rate of 15 mcg/kg for the first 2 h and then reduced to 5 mcg/kg/h for the following 4 h.

Endotoxemic shock was defined as a decrease in the systolic blood pressure of 30% of the baseline value [17]. When the shock was observed, a noradrenaline infusion was titrated to maintain a mean MAP > 65 mmHg.

All the animals were equally hydrated: during preparation phase an infusion of 30 ml/kg/h was administered and during the protocol the infusion was reduced to 20 ml/kg/h.

Ventilation was maintained in both groups the same as during the preparation phase (Vt 8 ml/kg, I:E 1:2, FIO_2_ 0.5, RR 25 cycles min^−1^ and PEEP 5 cmH_2_O). Arterial oxygen partial pressure (PaO_2_) was used to titrate the PEEP level: PaO_2_ was kept higher than 115 mmHg and PEEP was increased by 2 cmH_2_O every time the value was found below the threshold at the hourly check.

After 6 h of observation, three animals per group were transferred to the positron emission tomography–magnetic resonance imaging (PET–MRI) Research unit for image acquisition, the remaining animals were euthanized by an i.v. injection of a KCl bolus. The aim of the image acquisition was to measure perfusion and edema in the abdominal organs (such as small intestine, liver, spleen and kidneys). The animals examined with PET–MRI were killed once back in the laboratory, using the same method.

General anesthesia was maintained during the imaging acquisition while the endotoxin infusion was discontinued.

During autopsy, samples from the lungs (upper and lower lobes of both lungs), the duodenum, the colon (next to the ileocecal valve), the liver, the spleen and both kidneys were taken and used to measure edema (by wet–dry ratio) and inflammation (by immunohistochemistry or cytokine concentration measurement and histopathological analysis).

### Imaging techniques

Diffusion-weighted magnetic resonance imaging (DW-MRI) and PET imaging were performed on a clinical whole-body 3.0 T PET/MR system (Signa PET/MR, GE Healthcare, Waukesha, WI, USA).

DW-MRI is a technique that uses the diffusion of water molecules within tissues to assess as source of image contrast. In the presented study, using the bi-exponential intravoxel incoherent motion method (IVIM) [18], the diffusion coefficient (D; measure unit 10^−3^mm^2^/s) [19, 20] and the perfusion fraction (*f*) were estimated. D is the apparent diffusion coefficient corresponding to the magnitude of water molecular diffusion within tissues and *f* reflects the tissue volume fraction of capillaries, in which water molecular perfusion occurs.

A 10-min dynamic PET acquisition was performed after an i.v. bolus injection of oxygen-15 water ([^15^O]H_2_O). Regional values of perfusion (Flow) and volume of distribution (V_T_) (the partition coefficient of water in perfusable tissue) were analyzed.

For both techniques volumes of interest (VOIs) were drawn on the small intestine, the liver, the spleen and both kidneys for organ-specific analysis.

For detailed description of the imaging acquisition and analysis, see Appendix 2.

### Data collected

#### Hemodynamics and respiratory function data

Hemodynamic and respiratory measurements were continuously monitored and recorded at baseline and every hour during the 6-h observation period.

Following the World Society of the Abdominal Compartment Syndrome [21], IAP was measured through the bladder catheter by injecting a volume of 10 ml in the bladder (instead of the 25 ml volume recommended for human adults). The abdominal perfusion pressure was calculated as mean arterial pressure (MAP) minus IAP [22].

At baseline and each hour during the 6-h observation phase, arterial blood sample, and samples from pulmonary artery were collected for blood gas analyses (Radiometer 800, Copenhagen, Denmark).

#### Inflammation

Systemic inflammation was assessed by measuring cytokines (TNFα, IL6 and IL1b) concentration in arterial blood samples, taken at baseline and every hour during the experiment.

Abdominal inflammation was assessed by measuring TNFα, IL6 and IL1b concentration using ELISA in ascites and tissues samples, specifically the duodenum, the colon, the liver, the spleen and both kidneys.

A histopathological acute inflammation score from 0 to 5 [23] was assigned to samples from the same abdominal organs by the pathologist. The main features analyzed to produce the histopathological score were: the number and type of leukocytes, leukocyte localization, type, intensity, and extension of damage (necrosis, exfoliation, degeneration, apoptosis or erosion).

Samples from both lungs (upper and lower lobes) were also taken both for biochemical and histopathological assessment.

The biochemistry and the histopathological analyses were performed by persons blinded to the protocol.

#### Edema

To assess the global capillary leakage and edema, hemoglobin concentration in blood was used as a marker of hemoconcentration [24, 25].

Edema in single organs was assessed by three different methods.

First, the wet–dry weight [26] of tissue samples from both lungs (upper and lower lobes) duodenum, colon, liver, spleen and both kidneys was performed.

The samples were weighed fresh and subsequently placed in a 37° C oven for one week before re-weighed to obtain the dry weight. Data are presented as the ratio between the wet and the dry weight.

Furtherly, in the subgroup of animals undergoing the PET–MRI image acquisition, edema was also assessed by D (10^3^mm^2^/s) from DW-MRI (reflecting the magnitude of water diffusion within tissues) and by volume distribution (V_T_) from PET (reflecting the global amount of water within tissues) in the small intestine, liver, spleen and kidneys.

For intestine (duodenum and colon) and lung samples, edema was also assessed by the pathologist, who assigned a score to the samples, from 0 to 5 (0 = none, 1 = mild, 2 = moderate, 3 = severe; 4 = very severe; 5 = extreme). Besides, an assessment of the pulmonary atelectatic tissue (proportion over the entire sample scoring from 0 to 5; 0 = none, 1 = mild, 2 = moderate, 3 = extended; 4 = very extended; 5 = ubiquitous) was performed in the lungs’ samples.

#### Perfusion

Global perfusion was assessed by measuring the lactate concentration in arterial blood samples, at the baseline and every hour during the experiment.

Perfusion in several organs was assessed using DW-MRI and PET.

*f* from DW-MRI (representing the perfusion fraction) was assessed in VOIs drawn in the small intestine, the liver, the spleen, and the kidneys and thereafter compared between the groups. A corresponding comparison was performed using the PET Flow (ml/cm^3^/min).

### Statistical methods

Randomization was performed using Research Randomizer online software (http://www.rabdomizer.org). The software associates the progressive number of the animals to one of the two groups.

Parametric data (hemodynamics, PaO_2_, PaCO_2_, data from DW-MRI and PET) from the two groups were compared using the ANOVA test for repeated measurements and multiple t-tests with Bonferroni–Dunn correction. Cytokine and lactate concentrations were log-transformed and then analyzed as parametric data [27, 28].

Non-parametric data (wet–dry ratio and histopathological scores) were compared using the Mann–Whitney U-test.

The statistical features reported in the text or in the figures (mean and standard deviation or median and interquartile range) are always specified.

Correlations between parameters were evaluated using Pearson’s correlation coefficient.

A *p-value* < 0.05 was considered statistically significant.

The statistical analysis and the production of the figures were performed using GraphPad Prism software version 8.4.2 and R software version 3.6.3.

## Results

### Hemodynamic and respiratory function data

Nineteen piglets with an average weight of 30.1 (± 2.3) kg were included in the study. One pig died during the observation time following the hemodynamic shock and in another one, during the postmortem surgery, several abscesses were found; both pigs were excluded from the analysis. The rest of the animals were randomized to the Supine (*n* = 9) and the Prone (*n* = 8) groups.

In all the animals, a 30% reduction in the baseline value of the systolic pressure was observed within half an hour from the start of endotoxin infusion and interpreted as a sign of endotoxemic shock development.

PaO_2_/FiO_2_ decreased in both groups to a similar extent. The minimum value was reached after 4 h from the start of endotoxin infusion in the Supine group (240 ± 75 mmHg) and after 3 h in the Prone group (280 ± 89 mmHg). PEEP was found numerically higher (of about 0.5 cmH_2_O—nonsignificant) in the Supine group from the 4th hour after the start of the observation.

PaCO_2_ decreased faster in the Prone group (time x group interaction, *p-value* 0.007), but no other differences were identified in the respiratory parameters measured.

The hemodynamic parameters were similar in both groups at baseline and throughout the experiment (Table 1), except for the wedge pressure (PAOP) which was higher in the Prone group.

**Table 1:** Hemodynamics and respiratory data

	Baseline		1 h		2 h		3 h		4 h		5 h		6 h		ANOVA
	Supine	Prone	Supine	Prone	Supine	Prone	Supine	Prone	Supine	Prone	Supine	Prone	Supine	Prone	*p* value
PaO_2_ (KPa)	207 ± 53	207 ± 23	169 ± 53	200 ± 23	177 ± 38	146 ± 46	138 ± 46	153 ± 38	131 ± 38	153 ± 38	146 ± 38	169 ± 38	153 ± 38	169 ± 38	0.46
PaCO_2_ (KPa)	35 ± 6	36 ± 4	42 ± 6	40 ± 5	45 ± 8	43 ± 5	46 ± 8	42 ± 5	45 ± 8	38 ± 4	44 ± 7	40 ± 2	44 ± 6	39 ± 4	0.23 ⊥
PEEP (cmH_2_O)	5	5	5	5	5	5	5 ± 0.9	5 ± 1.1	5 ± 2	5 ± 1.4	5 ± 1.9	5 ± 1.5	5 ± 2.1	5 ± 1.5	–
Crs (mL/cmH_2_O)	27 ± 4	23 ± 3	21 ± 5	20 ± 2	19 ± 6	17 ± 4	18 ± 4	15 ± 2	18 ± 4	15 ± 2	16 ± 3	16 ± 1	17 ± 4	16 ± 1	0.15
EtCO_2_ (mmHg)	4.7 ± 0.7	4.9 ± 0.6	5.1 ± 1	5.6 ± 0.7	5.7 ± 0.8	6.3 ± 0.8	5.4 ± 0.6	5.8 ± 0.7	5.2 ± 0.8	5.6 ± 0.6	5.1 ± 0.9	5.5 ± 0.7	5.1 ± 0.9	5.6 ± 0.7	0.26
MAP (mmHg)	84 ± 11	91 ± 11	80 ± 16	88 ± 14	73 ± 13	81 ± 17	79 ± 16	83 ± 21	86 ± 22	91 ± 21	78 ± 23	87 ± 25	76 ± 19	77 ± 29	0.48
SAP (mmHg)	98 ± 18	104 ± 13	95 ± 15	102 ± 13	90 ± 18	99 ± 17	96 ± 18	106 ± 19	101 ± 17	103 ± 22	90 ± 21	96 ± 26	86 ± 23	96 ± 26	0.32
DAP (mmHg)	70 ± 14	79 ± 11	66 ± 16	77 ± 14	55 ± 15	67 ± 14	67 ± 17	72 ± 20	75 ± 23	81 ± 20	71 ± 22	74 ± 24	66 ± 19	67 ± 29	0.37
PAPm (mmHg)	18 ± 1	19 ± 4	31 ± 9	36 ± 7	34 ± 7	38 ± 1	39 ± 5	42 ± 8	38 ± 4	41 ± 9	36 ± 4	37 ± 9	33 ± 4	33 ± 10	0.41
PAPs (mmHg)	25 ± 4	26 ± 4	41 ± 7	45 ± 13	47 ± 9	51 ± 11	51 ± 8	55 ± 10	48 ± 6	51 ± 11	43 ± 5	47 ± 13	41 ± 3	42 ± 14	0.46
PAPd (mmHg)	12 ± 3	13 ± 5	25 ± 9	28 ± 8	27 ± 8	29 ± 3	33 ± 4	35 ± 7	33 ± 6	32 ± 9	30 ± 5	30 ± 10	29 ± 5	25 ± 11	0.91
PAOP (mmHg)	10 ± 2	12 ± 4	12 ± 4	16 ± 4	11 ± 3	14 ± 1	12 ± 4	14 ± 3	12 ± 2	15 ± 3	12 ± 3	14 ± 3	11 ± 3	13 ± 4	0.03*
Pulse (bpm)	96 ± 26	95 ± 24	118 ± 21	131 ± 42	131 ± 32	138 ± 45	140 ± 32	137 ± 37	129 ± 32	130 ± 34	118 ± 30	128 ± 35	119 ± 32	122 ± 30	0.75
CVP (mmHg)	8 ± 2	10 ± 4	9 ± 2	9 ± 3	8 ± 2	8 ± 3	9 ± 1	9 ± 4	9 ± 2	9 ± 3	9 ± 4	9 ± 3	11 ± 4	10 ± 3	0.99
IAP (mmHg)	6 ± 3	6 ± 2	7 ± 3	6 ± 2	7 ± 4	7 ± 2	9 ± 4	9 ± 2	9 ± 4	8 ± 3	9 ± 3	7 ± 3	10 ± 3	8 ± 4	0.44
APP (mmHg)	78 ± 11	85 ± 12	73 ± 14	81 ± 13	66 ± 11	74 ± 16	71 ± 16	75 ± 23	77 ± 22	83 ± 21	71 ± 25	80 ± 26	66 ± 20	69 ± 32	0.41
CO (L/min)	3.1 ± 0.6	3 ± 0.7	3.4 ± 0.7	3.1 ± 0.8	3.4 ± 0.7	3.4 ± 0.9	2.8 ± 0.9	2.5 ± 0.4	2.4 ± 0.6	2.3 ± 0.3	2.3 ± 0.7	2.2 ± 0.4	2.1 ± 0.6	2.1 ± 0.4	0.65
Urine Out (ml/kg/h)													3.4 ± 0.8	2.4 ± 0.8	0.01*

MAP = mean arterial pressure; SAP = systolic arterial pressure; DAP = diastolic arterial pressure; PAPm = mean pulmonary artery pressure; PAPs = systolic pulmonary artery pressure; PAPd = diastolic pulmonary artery pressure; CVP = central venous pressure; PAOP = pulmonary artery occlusion pressure; IAP = intra-abdominal pressure; APP = abdominal perfusion pressure; CO = cardiac output; EtCO_2_ = end-tidal carbon dioxide; PEEP = positive end-expiratory pressure; Crs = compliance of the respiratory system; PaO_2_ = arterial partial pressure of oxygen; PaCO_2_ = arterial partial pressure of carbon dioxide. All the measurements are reported as mean ± standard deviation, except for PEEP which is reported as median and min–max range. In the last column on the right, the *p* value of the ANOVA test for the group factor is reported. A *p*-value < 0.05 is accompanied by *, while a time x group difference (*p* < 0.05) is represented by ⊥ (the *p* value is not reported in the table, but in the text)

The infusion rate of noradrenaline (used to maintain MAP > 65 mmHg) during the observation period was similar in both groups (median [IQR1–IQR3]. Supine group: 0.072 [0.070–0.075]; prone group: 0.068 [0.065–0.073] μg kg^−1^ min^−1^). IAP (intra-abdominal pressure) was also similar.

The amount of crystalloid infused in both groups during the preparation phase and during the observation period did not differ (median [IQR1–IQR3]. Supine group: 1350 [1300–1450]; Prone group: 1350 [1250–1400] ml).

Urinary output was higher in the Supine group by 30% (see Table 1).

### Inflammation

**Fig. 1:**
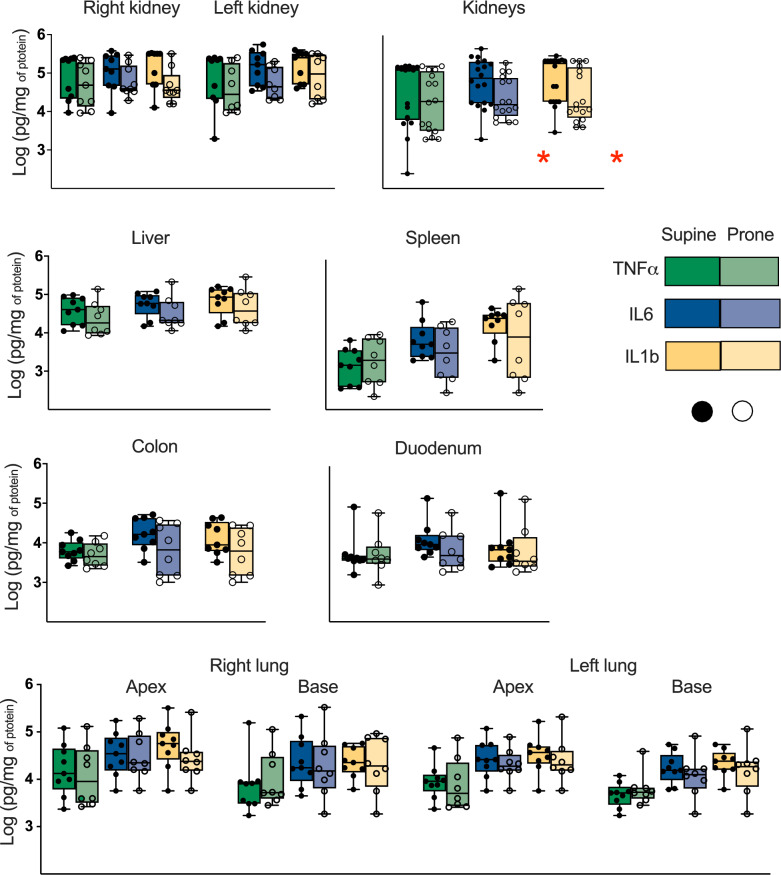
Cytokines concentration in tissue samples of abdominal organs and lungs. Every graph reports the Log10 (pg/mg of protein) concentration of TNFa (green), IL6 (blue) and IL1b (yellow) in the tissue sample of a specific organ. The two kidneys are represented separately as well as together (the individual data for separate kidneys are added and shown in the same graph s bar); the two lungs are represented separately, as well as apex and base of each lung. The boxes represent the median and interquartile range, while the whiskers show minimum and maximum values. Individual values per subject are also reported and represented by dots. Significant differences between the groups (found with corrected t-test) are marked with a red star. See the figure legend for grouping (Supine = 9 subjects; Prone = 8 subjects)

**Fig. 2:**
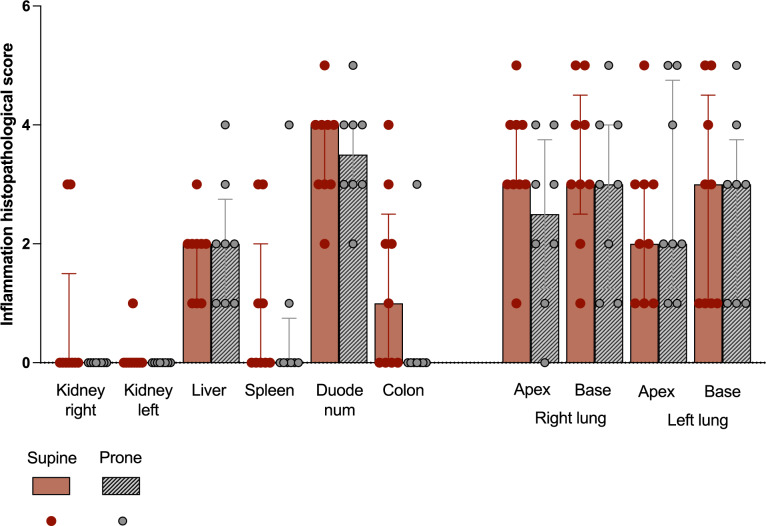
Inflammatory histopathological score in abdominal organs and lungs. The score (0 to 5) given by the pathologist to the tissue sample of each subject is reported in the figure. The two kidneys and lungs are represented separately; the apex and base of the lungs are also represented separately. Bars represent median and whiskers report second and third interquartiles. The individual subject’s score is reported as dots. See the figure legend for grouping (supine = 9 subjects; prone = 8 subjects)

IL6 and IL1b were higher in the kidney tissues of the Supine group. No other differences in cytokine concentrations were found in blood, ascites or tissue samples. No differences were found in the inflammation histopathological score of the investigated organs. Figure 1 reports cytokine concentrations and Fig. 2 the inflammation histopathological score in tissue samples.

### Edema

Hemoglobin increased in both groups over the 6-h observation period with no difference between the groups. The mean ± SD at baseline was 19.4 ± 4.9 g/dL in the Supine group and 18.4 ± 3.3 g/dL in the Prone group; after the 6-h observation period, it was 23.9 ± 5.2 g/dL in the Supine group and 22.2 ± 6.6 g/dL in the Prone group.

**Fig. 3:**
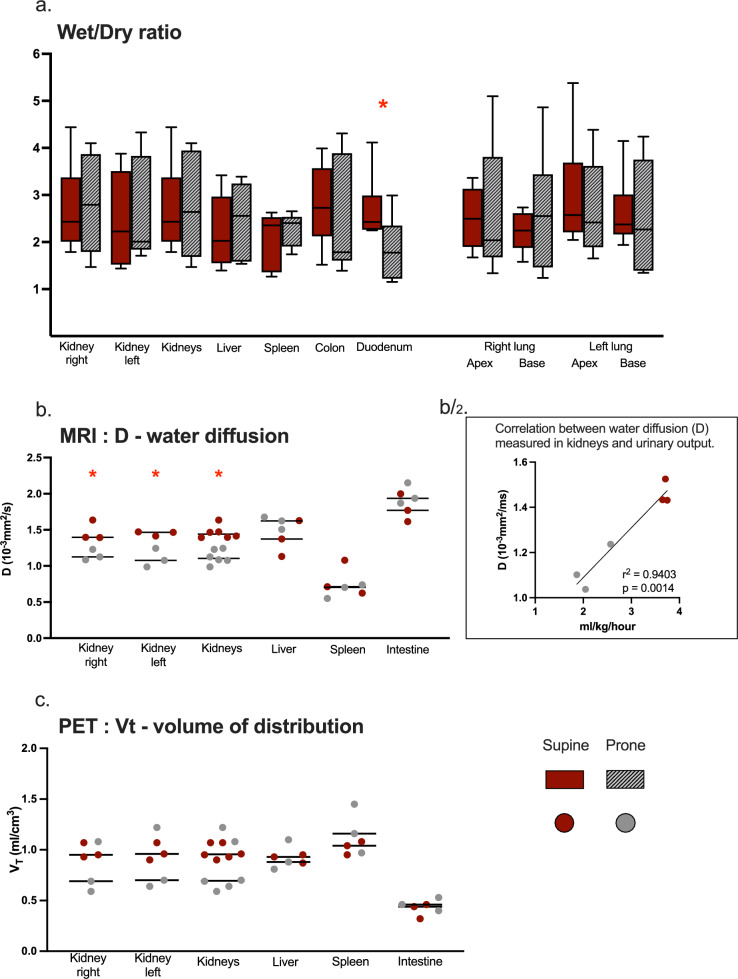
Edema assessment in abdominal organs and lungs. **a** Wet/dry ratio (adimensional) calculated in tissue samples of different organs. Kidneys are represented separately as well as together (the individual data for separate kidneys are added and shown in the same graph s bar). Lungs are also represented separately, as well as the apex and base of both lungs. Boxes represent interquartile ranges and the line in the box is the median, whiskers represent minimum and maximum values range. **b** D (or apparent diffusion coefficient of water) measured with DW-MRI as a surrogate for edema in the region of interest (ROIs) of different abdominal organs. Kidneys are represented separately as well as together. Individual values and mean values (as a black line) are represented. b/2. Correlation between D (or water diffusion—10^−3^mm^2^/s) measured in kidneys and urinary output (ml/kg/h). For the correlation, the mean value of D in the right and left kidney has been calculated for each subject and used for the analysis. **c** V_T_ (or volume of distribution—ml/cm^3^) measured with PET as a surrogate for edema in the region of interest (ROIs) of different abdominal organs. Kidneys are represented separately as well as together. Individual values and mean values (as a black line) are represented. Significant differences between the groups (found with Mann–Whitney U-test for the wet/dry ratio and with corrected t-test for the imaging data) are marked with a red star. See figure legend for grouping: (for wet/dry ratio: Supine = 9 subjects; Prone = 8 subjects; for imaging data: Supine = 3 subjects; Prone = 3 subjects)

Wet–dry weight showed higher water content in the duodenum of the Supine group, while D (obtained from DWI—magnitude of water diffusion) was higher in the kidneys of the Supine group. D value correlated with the urinary output (for the right kidney r^2^ = 0.81; for the left kidney r^2^ = 0.93); see Fig. 3.

No differences in V_T_ (volume of distribution—obtained from the PET imaging analysis as a surrogate for the water content) were found in any of the investigated organs.

Figure 3 resumes the organs’ edema assessment.

The pathologist reported enhanced edema and atelectatic tissue in the lower lobe of the left lung of the Supine group. The rest of the lung samples showed similar grades of atelectasis in both groups, with a gradient from apex to base. Table 2 reports the pathologist’s edema and atelectasis assessment.

**Table 2:** Histopathological assessment of edema and atelectatic tissue in samples from the apex and base of both lungs

		Right lung Apex	Right lung Base	Left lung Apex	Left lung Base
Edema	Supine	3 (1; 4)	3 (3; 4)	1 (0; 4)	4 (4; 5)
	Prone	2,5 (1,5; 3)	2,5 (1; 3,25)	3 (2,5; 4,25)	3 (2; 4)
Atelectasis	Supine	2 (0; 3)	4 (2; 5)	2 (0; 3)	4 (4; 5)
	Prone	3 (2; 4)	2.5 (1.75; 4)	2 (1; 4)	3 (1; 4)

The pathologist assessed both features giving a score from 0 to 5 (0 = none, 1 = mild, 2 = moderate, 3 = severe; 4 = very severe; 5 = extreme/ubiquitous)

### Perfusion

Lactate concentration was similar in both groups [maximum value measured (Log10 of the concentration—μg ml^−1^) supine group: 0.6 ± 0.21; prone group: 0.52 ± 0.16)].

Perfusion was found higher by both DWI (f value) and PET (Flow) in the kidneys of the Supine group. *f* correlated with flow (r^2^ 0.7; *p-value* < 0.01) for both kidneys.

**Fig. 4:**
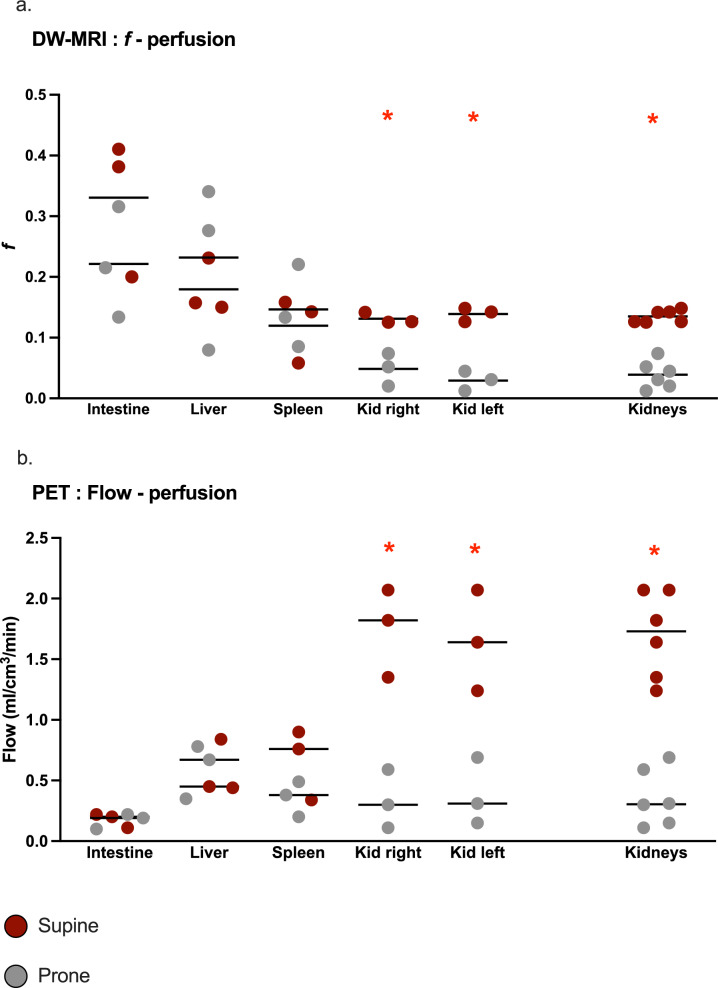
Perfusion in abdominal organs. **a** f value (adimensional) measured with DW-MRI as an assessment of perfusion. Individual values are represented as dots and mean values per group as horizontal lines. **b** Flow (ml/cm^3^/min) measured with PET as an assessment of perfusion. Individual values are represented as dots and mean values per group as horizontal lines. See the figure legend for grouping (Supine = 3 subjects; Prone = 3 subjects). Significant differences between the groups (found with corrected t-test) are marked with a red star

Figure 4 resumes organs’ perfusion assessment.

**Fig. 5:**
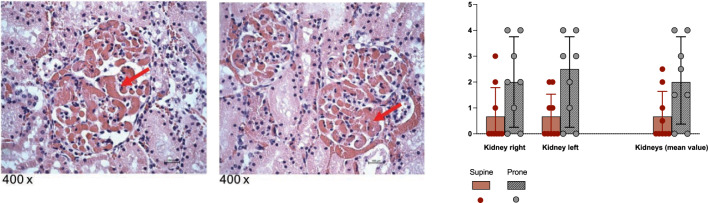
Glomerular thrombosis. On the left side: two pictures of the kidney samples taken by the pathologist in two different subjects belonging to the prone group (magnification 400 ×, coloration hematoxylin–eosin) that show thrombosis in glomeruli (arrows). On the right side: the glomerular thrombosis score (0 to 4) given by the pathologist to the kidney tissue samples. The two kidneys are represented separately as well as together (a mean value of the score given to the right and left kidney has been used for analysis and it is shown in the right part of the graph). See the figure legend for grouping (supine = 9 subjects; prone = 8 subjects)

The evaluation of glomerular thrombosis was decided following the accidental observation of an enhanced incidence in the kidneys of the prone group’s subject. A score was given to each subject’s sample, from 0 to 4 (0 = no thrombosis; 1 = mild thrombosis; 2 = moderate thrombosis; 3 = severe, spread thrombosis; 4 = massive thrombosis). See Fig. 5.

## Discussion

This study compared abdominal organ's edema, perfusion, and inflammation in supine and prone positions during respiratory failure. We found reduced renal perfusion and urine output, associated with enhanced glomerular thrombosis, in prone-positioned animals.

This study is the first to present MRI and PET-assessed abdominal organ perfusion and edema in supine vs prone positioning for respiratory failure. The endotoxemic model induced a reduction in PaO_2_/FiO_2_ comparable to mild ARDS. Similar oxygenation and perfusion pressure were maintained in both groups, allowing the assessment of abdominal organs without the risk of confounding factors.

The two groups were ventilated similarly, using protective lung ventilation [29] (Vt 8 ml/kg). Compliance of the respiratory system did not vary between the groups, but it decreased after the induction of endotoxemia, indicating endotoxin-induced lung damage similar to that of a mild-to-moderate ARDS (minimum PaO_2_/FiO_2_ was about 200). In the Prone group PaCO_2_ decreased faster and the histopathological analysis showed less edema and atelectasis in the lower lobes of the lungs. These results align with most studies reporting superior ventilation–perfusion match when prone positioning [30] is used.

Body position did not impact MAP and cardiac output. However, it did determine a higher wedge pressure in the prone group, unlike a small study that reported a reduction in the right heart load associated with pronation [31].

IAP was similar in the two groups and no signs of abdominal hypertension were detected in the Prone group.

Looking at abdominal organs, liver, spleen, and colon showed similar degrees of edema and inflammation in both groups, as well as no difference in perfusion. Duodenum was found more edematous in the Supine group when water content was measured by wet/dry ratio; the position of the animal body could have changed the lymphatic and venous drainage of the organ, which is the most sensitive to changes in perfusion and lymphatic drainage [32, 33]. The difference was not detected by the pathologist who assessed the small intestine samples. The small intestine is an extended organ, proven to have inhomogeneous perfusion and edema during endotoxemia, which could have contributed to inconclusive results.

Kidneys’ perfusion was less than half in the Prone group compared to the Supine group according to both DW-MRI and PET results. D, representing the magnitude of water diffusion within tissues, was also found higher in the supine group. In the kidneys, D is generally correlated to GFR [34–36] and urinary output, and should therefore not be interpreted exclusively as an assessment of edema, but as depending on perfusion changes as well. This hypothesis is confirmed by the lack of difference in edema measured by wet–dry weight and PET V_T_.

Glomerular thrombosis is not a new finding in the kidneys of animals exposed to endotoxin, but the abundance of it noticed and reported by the pathologist in pronated animals is an uncommon finding. It could be the cause of the low perfusion and subsequently reduced filtration rate. A link between prone position and increased deep venous thrombosis was previously described in a study by Gebhard et al. [16], but no other studies previously investigated glomerular thrombosis in pronated animals or patients. Alternatively, the thrombosis could be the result of a slow flow in the glomerular vessels. No other signs of increased thrombosis were reported in any other abdominal organs.

IL6 and IL1b concentrations in the kidneys were found lower in the Prone group. This could be due to the extremely low perfusion that partially protects the kidneys from the action of the cytokines in the bloodstream. In a previous study [32], a reduction in renal perfusion produced a higher inflammation over a similar observation period, but the decrease in perfusion was not as pronounced as in the presented study.

The low perfusion rate associated with the widespread glomerular thrombosis seen in the kidneys of the Prone group could be harmful to renal function in the long term. Further research is needed to ascertain these findings.

DW-MRI [18] and PET were used to investigate edema and perfusion [19] in abdominal organs. The use of DW-MRI in abdominal organs has grown in recent years [37]. Still, its application to an endotoxemic model, to determine edema and perfusion of entire organs instead of specific lesions is novel, although the efficacy of the methodology was demonstrated in multiple studies [38, 39]. [^15^O]H_2_O PET was used as a control method for DW-MRI for perfusion and edema assessment.

A direct comparison between results obtained with DW-MRI and PET is impossible due to the difference in the VOI size dimensions and the different nature of the parameters derived from the two techniques. Considerably larger VOIs were drawn for the PET data analysis with the intent of including as much organ tissue as possible to capture its global perfusion. DW-MRI VOIs were instead smaller, as it was crucial to exclude for example large vessels and intestine content in the analyzed volume.

This can be the reason for the difference observed in intestinal perfusion and edema between the two techniques: DW-MRI VOIs were small and probably failed to capture the global perfusion assessed by PET, while instead representing regional perfusion which, during endotoxemia, can show high variability.

Besides, *f* represents the fractional volume where perfusion occurs and not the perfusion per se; while the Flow from PET is a direct measurement of the tissue perfusion. Otherwise, *f* correlated with Flow in kidneys, showing that DW-MRI is effective in capturing variations of renal perfusion.

Further studies are needed to compare the two techniques in larger groups, but the results in kidneys are promising.

### Limitations

There are some limitations that need to be addressed.

First, the selected animal model could have produced results that might be difficult to transfer to a clinical setting, as the body position of the animals differs from that of humans. Otherwise, the porcine model has been largely used for ventilation studies, including studies on pronation [40].

The study is a model of endotoxemic-related lung injury, and the results need to be confirmed in different lung injury models (such as the VILI-model).

The small number of subjects included, especially in the imaging data analysis, needs to be addressed as a limitation. The study was conducted following the 3R principle for animal research and considering the high cost of the imaging technique included.

The presented protocol was short, only lasting six observation hours; more extended pronation time could have produced different results. Otherwise, the intent of the study was to investigate the effect of pronation on abdominal organs in the short term and in many clinical settings 6 h represents the minimum time for a pronation session. Furthermore, the pathological alterations in the kidneys of the pronated subjects are particularly interesting because they occurred in such a short time.

The decision to build a mild–moderate ARDS model was driven by the recent tendency to apply pronation to less severe cases than in the past, a trend that characterized the COVID-19 pandemic. Besides, the mild lung damage obtained reduces the risk of confounding factors related to altered tissue oxygenation or CO_2_ retention.

## Conclusions

The prone position in this porcine ARDS model was associated with glomerular thrombosis and low renal perfusion, with related low urinary output.

## Supplementary Information

Additional file 1.

Additional file 2.

## Data Availability

The datasets used and/or analyzed during the current study are available from the corresponding author on reasonable request.

## Abbreviations

ARDS: Acute respiratory distress syndrome
APP: Abdominal perfusion pressure
COVID 19: Coronavirus disease 19
D: Apparent diffusion coefficient
DW-MRI: Diffusion-weighted magnetic resonance imaging
ELISA: Enzyme-linked immunosorbent assay
*f*: Perfusion fraction
IAP: Intra-abdominal pressure
IL1b: Interleukin 1 b
IL6: Interleukin 6
MAP: Mean arterial pressure
MRI: Magnetic resonance imaging
PEEP: Positive end-expiratory pressure
PET: Positron emission tomography
SARS-CoV-2: Severe acute respiratory syndrome coronavirus 2
TNFα: Tumor necrosis factor α
V_T_: Volume of distribution
Vt: Tidal volume
