# Supplementary material for: Enhanced glomerular thrombosis in pronated animals with ARDS
Source: Intensive Care Med Exp. 2025 Mar 20;13:36. doi: 10.1186/s40635-025-00747-7 (PMC11926287; doi:10.1186/s40635-025-00747-7)
Supplement: Supplementary file 1 — Additional file 1. [file 40635_2025_747_MOESM1_ESM.docx]

***Appendix 1: Preparation***

Animals were pre-medicated with Zoletil Forte (tiletamine and zolazepam) 6 mg kg^-1^ and Rompun (xylazine) 2.2 mg kg^-1^ intramuscularly. After 5 to 10 minutes, the animals were placed supine on an operating table and monitored with an EKG and a SpO_2_ probe. An intravenous (i.v.) bolus of fentanyl 10-20 µg kg^-1^ was given and a tracheotomy was performed with the insertion of an 8 mm diameter endotracheal tube (Mallinckrodt Medical, Ireland).

Ventilation was started in volume-control mode by a Servo-I ventilator (Maquet, Sweden) with a tidal volume (V_T_) of 8 mL kg^-1^, inspiratory:expiratory ratio (I:E) of 1:2, fraction of inspired oxygen (FIO_2_) of 0.5, respiratory rate (RR) 25 cycles min^-1^ and PEEP 5 cmH_2_O for the entire preparation period.

Anesthesia was maintained with a continuous i.v. infusion of ketamine 30 mg kg^-1^ h^-1^, midazolam 0.1 mg kg^-1^ h^-1^ and fentanyl (0.3 mg kg^-1^ h^-1^). After checking the adequacy of the anesthesia to prevent responses to painful stimulation, muscle relaxation was added as a continuous i.v. infusion of rocuronium 3 mg kg^-1^ h^-1^ after a loading bolus of 40 mg.

A triple-lumen, thermistor-tipped balloon catheter (Swan-Ganz catheter, 7 Fr) was placed in the pulmonary artery from the right external jugular vein. Positioning of a central venous catheter was performed through the same access route. A neck artery was cannulated. A second triple-lumen, thermistor-tipped balloon catheter was introduced into the hepatic vein from the left internal jugular vein (positioning was ascertained by fluoroscopy) and a 4 Fr catheter in the portal vein via the splenic vein. To introduce the catheter in the splenic vein, a laparotomy was performed to expose the spleen. The splenic vein was cannulated with an 18 G peripheral catheter and a 4 Fr catheter inserted over a metal guidewire using the Seldinger technique. The spleen was carefully repositioned in the abdomen and the peritoneum, muscle layers and skin were sutured. The catheters were used for blood sampling and pressure measurements. Cardiac output was measured by thermodilution using the thermistor-tipped catheter. A bladder catheter was inserted to collect urine and to measure IAP.

Abdominal perfusion pressure (APP) was calculated as:

MAP (mean arterial pressure) – IAP.

Table 1 includes a detailed description of the tissue sampling.

| **Immunohistochemistry** – *ELISA test dosing TNFα, IL6 and IL1b* | |
| --- | --- |
| ***Organ*** | ***Description of sample*** |
| Duodenum | 3-5 mm^3^ tissue sample, from around 1 cm from the esophageal lower sphincter. Rinse in water to eliminate the residual gastric secretion/bile. |
| Ileum | 3-5 mm^3^ of ileum, next to the ileocecal valve. Rinse in water to eliminate the residual feces. |
| Liver | 5-7 mm^3^ tissue sample from the 5^th^ lobe (below the gallbladder). |
| Spleen | 5-7 mm^3^ tissue sample from the peripheric portion of the organ’s tail. |
| Kidneys | 5-7 mm^3^ sample from the pole cortex. |
| Lung (upper lobe) | 3-5 mm^3^ tissue sample from the upper dorsal lobe of both lungs |
| Lobe (lower lobe) | 3-5 mm^3^ tissue sample from the upper ventral lobe of both lungs |
| **Wet-dry ratio** | |
| ***Organ*** | ***Description of sample*** |
| Duodenum | 1-2 cm tissue sample, from around 1 cm from the esophageal lower sphincter. Rinsed in water to eliminate the residual gastric secretion/bile. |
| Ileum | 1-2 cm of ileum, next to the ileocecal valve. Rinsed in water to eliminate the residual feces. |
| Liver | 2-3 cm^3^ tissue sample from the 5^th^ lobe (below the gallbladder). |
| Spleen | 2-3 cm^3^ tissue sample from the peripheric portion of the organ’s tail. |
| Kidneys | 2-3 cm^3^ sample from the pole cortex. |
| Lung (upper lobe) | 1-2 cm^3^ tissue sample from the upper dorsal lobe of both lungs |
| Lobe (lower lobe) | 1-2 cm^3^ tissue sample from the upper ventral lobe of both lungs |
| **Histopathological analysis** | |
| ***Organ*** | ***Description of sample*** |
| Duodenum | 1-2 cm tissue sample, from around 2-3 cm from the esophageal lower sphincter. Rinsed in water to eliminate the residual gastric secretion/bile. |
| Ileum | 1-2 cm^3^ of ileum, next to the ileocecal valve. Rinsed in water to eliminate the residual feces. |
| Liver | 2-3 cm^3^ tissue sample from the 5^th^ lobe (below the gallbladder). |
| Spleen | 2-3 cm^3^ tissue sample from the peripheric portion of the organ’s tail. |
| Kidneys | 2-3 cm^3^ sample from the pole cortex. |
| Lung (upper lobe) | 1-2 cm^3^ tissue sample from the upper dorsal lobe of both lungs |
| Lobe (lower lobe) | 1-2 cm^3^ tissue sample from the upper ventral lobe of both lungs |

Table 1. List of organs’ samples taken by the method of analysis.
