# Supplementary material for: Enhanced glomerular thrombosis in pronated animals with ARDS
Source: Intensive Care Med Exp. 2025 Mar 20;13:36. doi: 10.1186/s40635-025-00747-7 (PMC11926287; doi:10.1186/s40635-025-00747-7)
Supplement: Supplementary file 2 — Additional file 2. [file 40635_2025_747_MOESM2_ESM.docx]

***Appendix 2*: *Imaging***

DW-MRI and PET were performed on a clinical whole-body 3.0 T PET/MR system (Signa PET/MR, GE Healthcare, Waukesha, WI, USA).

*DW-MRI*

DW-MRI was performed with a two-dimensional (2D) single-shot spin-echo echo-planar imaging sequence using an upper anterior array coil and the following scan parameters: axial acquisition, respiratory triggering, field of view (FOV, right-left × anterior-posterior) 300 × 300 mm^2^ (the FOV was increased to 320 × 320 mm^2^ for three pigs in order to fully cover the abdominal wall), 36 slices, slice thickness 8 mm, acquired/reconstructed matrix = 96 × 128 / 256 × 256, repetition time 6667 - 15000 ms, echo time 70.8 ms, three diffusion sensitizing directions with *b*-values 0, 20, 50, 75, 100, 200, 300, 600, 900 s/mm^2^.

Using the bi-exponential intravoxel incoherent motion (IVIM)(1) method, the apparent diffusion coefficient (D; measure unit 10^-3^mm^2^/s(2)^,^(3) and the perfusion fraction (*f*) were estimated. D corresponds to the magnitude of water molecular diffusion within tissues and *f* reflects the tissue volume fraction of capillaries, in which water molecular perfusion occurs.

All measurements were performed in volumes of interest (VOIs) manually outlined in five organs using the medical imaging post-processing software Olea Sphere 3.0-SP26: liver, kidney cortex (left and right), small intestine, and spleen. The VOI mean value and standard deviation of D and *f* were estimated from the parametric maps of D and *f* assessed using the same software.

The goodness of fit, by means of the coefficient of determination (R^2^), was considered by only including voxels with R^2^ > 0.8 in the VOIs.

*PET imaging*

An i.v. bolus injection of 400 MBq of oxygen-15 water ([^15^O]H_2_O) was administered when the MR acquisition was completed, and followed by one 10 ml bolus of NaCl at 0.8 ml/s and one 30 ml bolus at 2 ml/s. The [^15^O]H_2_O effective dose was 0.4 mSv. A 10-min dynamic PET acquisition was performed consisting of 28 frames of increasing duration (12×5, 3×10, 2×15, 3×20, 2×30, 6×60 sec). A 128×128 image matrix and a 300 mm axial FOV were used, and images were reconstructed using BSREM with β-value 300.

VOIs in the aorta and the organs of interest (liver, spleen, intestine, and kidneys) were manually delineated on PET using Hermes (Hybrid Viewer PDR 6.13, Hermes Medical Solutions) and Voiager software (VOIager 4.0.5). Kinetic modeling was performed in Matlab (Matlab R2018b) using a single-tissue compartment model(4) with an image-derived arterial input function. A dual-input single-tissue compartment model with portal vein input function modelled as a delayed and dispersed arterial input function was used for the liver(5)^,^(6). Regional values of perfusion (Flow) and volume of distribution (V_T_) (the partition coefficient of water in perfusable tissue) were analyzed for the organs of interest with the addition of portal vein perfusion fraction for the liver.

*Volumes of interest (VOIs)*

On DW-MR images, one VOI for each target organ was drawn in the six selected animals.

The average size of the VOIs in cm^3^ was (median (IQR1 – IQR3)): 6.53 (5.8 – 7.05) for the liver; 2.69 (2.61 – 2.76) for the intestine; 3.95 (2.86 – 4.1) for the spleen; 3.68 (3.54 – 3.83) for the right kidney; and 3.51 (3.47 – 3.71) for the left kidney.

New VOIs were drawn for PET data analysis, one for each target organ.

The average size of the VOIs in cm^3^ was (median (IQR1 – IQR3)): 101.2 (83.4 – 122.4) for the liver; 168 (132.9 – 178.6) for the intestine; 8.5 (7.2 – 9.1) for the spleen; 35.6 (26.3 – 38.4) for the right kidney; and 30.4 (26.5 – 32.3) for the left kidney.

**Bibliography**

1. Koh D-M, Collins DJ, Orton MR. Intravoxel incoherent motion in body diffusion-weighted MRI: reality and challenges. AJR Am J Roentgenol. 2011 Jun;196(6):1351–61.

2. Andreou A, Koh DM, Collins DJ, Blackledge M, Wallace T, Leach MO, et al. Measurement reproducibility of perfusion fraction and pseudodiffusion coefficient derived by intravoxel incoherent motion diffusion-weighted MR imaging in normal liver and metastases. Eur Radiol. 2013;23(2):428–34.

3. Taouli B, Beer AJ, Chenevert T, Collins D, Lehman C, Matos C, et al. Diffusion-weighted imaging outside the brain: Consensus statement from an ISMRM-sponsored workshop. J Magn Reson Imaging. 2016 Feb;

4. Kety SS, Schmidt CF. THE NITROUS OXIDE METHOD FOR THE QUANTITATIVE DETERMINATION OF CEREBRAL BLOOD FLOW IN MAN: THEORY, PROCEDURE AND NORMAL VALUES. J Clin Invest. 1948 Jul;27(4):476–83.

5. Ziegler SI, Haberkorn U, Byrne H, Tong C, Kaja S, Richolt JA, et al. Measurement of liver blood flow using oxygen-15 labelled water and dynamic positron emission tomography: limitations of model description. Eur J Nucl Med. 1996 Feb;23(2):169–77.

6. Slimani L, Kudomi N, Oikonen V, Jarvisalo M, Kiss J, Naum A, et al. Quantification of liver perfusion with [(15)O]H(2)O-PET and its relationship with glucose metabolism and substrate levels. J Hepatol. 2008 Jun;48(6):974–82.
